# Eye Movement Deficits Are Consistent with a Staging Model of pTDP-43 Pathology in Amyotrophic Lateral Sclerosis

**DOI:** 10.1371/journal.pone.0142546

**Published:** 2015-11-11

**Authors:** Martin Gorges, Hans-Peter Müller, Dorothée Lulé, Kelly Del Tredici, Johannes Brettschneider, Jürgen Keller, Katharina Pfandl, Albert C. Ludolph, Jan Kassubek, Elmar H. Pinkhardt

**Affiliations:** 1 Department of Neurology, University of Ulm, Ulm, Germany; 2 Section Clinical Neuroanatomy, Department of Neurology, University of Ulm, Ulm, Germany; 3 Center for Neurodegenerative Disease Research (CNDR), University of Pennsylvania School of Medicine, Philadelphia, PA, United States of America; Tokai University, JAPAN

## Abstract

**Background:**

The neuropathological process underlying amyotrophic lateral sclerosis (ALS) can be traced as a four-stage progression scheme of sequential corticofugal axonal spread. The examination of eye movement control gains deep insights into brain network pathology and provides the opportunity to detect both disturbance of the brainstem oculomotor circuitry as well as executive deficits of oculomotor function associated with higher brain networks.

**Objective:**

To study systematically oculomotor characteristics in ALS and its underlying network pathology in order to determine whether eye movement deterioration can be categorized within a staging system of oculomotor decline that corresponds to the neuropathological model.

**Methods:**

Sixty-eight ALS patients and 31 controls underwent video-oculographic, clinical and neuropsychological assessments.

**Results:**

Oculomotor examinations revealed increased anti- and delayed saccades’ errors, gaze-palsy and a cerebellary type of smooth pursuit disturbance. The oculomotor disturbances occurred in a sequential manner: *Stage 1*, only executive control of eye movements was affected. *Stage 2* indicates disturbed executive control plus ‘genuine’ oculomotor dysfunctions such as gaze-paly. We found high correlations (*p*<0.001) between the oculomotor stages and both, the clinical presentation as assessed by the ALS Functional Rating Scale (ALSFRS) score, and cognitive scores from the Edinburgh Cognitive and Behavioral ALS Screen (ECAS).

**Conclusions:**

Dysfunction of eye movement control in ALS can be characterized by a two-staged sequential pattern comprising executive deficits in Stage 1 and additional impaired infratentorial oculomotor control pathways in Stage 2. This pattern parallels the neuropathological staging of ALS and may serve as a technical marker of the neuropathological spreading.

## Introduction

Amyotrophic lateral sclerosis (ALS) is the most common age-related motorneuron disease characterized by a rapidly progressive neurodegenerative disorder with involvement of the upper and lower motoneurons, leading to death with a mean survival of approximately 3 years [1]. Nonetheless, the underlying pathology manifests beyond motoneuron degeneration, affecting also widespread extramotor areas. Subtle cognitive deficits, with predominant involvement of executive function, are more common in ALS than previously thought [1] even in non-demented ALS patients.

As previously described [2], ALS progresses according to a sequential regional pattern that can be defined by four neuropathological stages with primary affection of the cerebral cortex and dissemination by involved cortical projection neurons, along corticofugal axons. These findings raise the issue of whether the apparent neuropathological progression can be paralleled by clinical staging. However, subtle non-motor changes may be beyond the clinical threshold, and minor extra-pyramidal motor (e.g., cerebellar) symptoms may be masked by the severity of anterior horn cell and pyramidal pathology.

Oculomotor abnormalities are not regarded as a predominant symptom in ALS. However, in recent years an increasing number of studies have indicated a broad range of eye movement deficits in these patients [3–5]. The brain network pathology underlying oculomotor deficits is well known and comprises widespread influences from the brainstem to higher cortical regions that are crucial for saccade generation and smooth pursuit [6,7]. In particular, however, it is only when the neuropathological spreading suggested by Brettschneider et al. [2] is taken into account that the multitude of eye movement deficits can be properly ascribed to its pathological cause in ALS. In the present study, we systematically characterized oculomotor disturbances in 68 ALS patients (50 with spinal and 18 with bulbar onset) in comparison to 31 controls and described the underlying network pathology as defined in the sequential pattern of the four ALS stages. Then, we investigated if eye movement deterioration in ALS can be categorized within a staging system of oculomotor decline that corresponds to the neuropathological stages of ALS [8]. We assumed two stages with stage 1 comprising executive deficits of oculomotor control and stage 2 with additional pathology in the brainstem circuitry for saccade generation (reticular formation and precerebellary nuclei).

## Materials and Methods

### Subjects and clinical characterization

Statistics of detailed clinical characteristics and demographic features of all participants are summarized in Table 1. Recordings of eye movements (for details of the protocols, see *Recording of eye movements*) from 68 ALS-patients including 50 patients with spinal and 18 with bulbar disease onset and 31 age- and gender-matched healthy individuals in addition to neuropsychological and cliniconeurological assessments were obtained during routine clinical examination. Both patient subgroups matched with respect to age, gender, duration of disease, age of onset, education, and revised ALS Functional Rating Scale (ALSFRS-R) score (Table 1). All patients with ALS were included in the German Motor Neuron Disease Network and had given their written informed consent according to institutional guidelines, as approved by the Ethics Committee of the University of Ulm.

**Table 1:** Subject demographics and clinical characterization. Data are shown as median (interquartile range), min-max.

	Healthy controls (*N* = 31)	ALS, All (*N* = 68)	*p*-value	ALS, spinal onset (*N* = 50)	ALS, bulbar onset (*N* = 18)	*p*-value
**Gender** (male:female)	**14: 17**	**38: 30**	**0.388** ^a^	**14: 17**	**38: 30**	0.535^b^
**Age** (years)	**56** (48–64), 31–77	**61** (54–70), 31–80	0.167^c^	**62** (54–70), 33–80	**59** (48–67), 31–76	0.232^d^
**Duration of disease** (month)	NA	**13 (**9–23), 3–64	NA	**13** (9–22), 3–49	**13** (7–27), 5–64	0.867^c^
**Age of onset** (years)	NA	**59** (51–69), 30–80	NA	**60** (52–69), 31–80	**55** (46–65), 30–74	0.303^c^
**ALSFRS-R** ^**e**^	NA	**40** (36–42), 15–47	NA	**40** (37–43), 15–47	**38** (32–42), 23–45	0.078^c^
**Years of education**	**14** (13–16), 9–19	**12** (11–14), 6–21	**0.015** ^c^	**13** (11–15), 6–21	**13** (11–14), 10–20	0.051^e^
**ECAS** ^**f**^	**116** (108–122), 90–126	**106** (98–115), 58–132	**0.001** ^c^	**106** (98–112), 77–125^†^	**108** (87–119), 58–132	**0.005** ^e^
**El Escorial criteria** (possible:probable:definite)	NA	**2: 11: 55**	NA	**2: 9: 39**	**0: 2: 16**	NA

^a^Fisher’s exact test refers to comparison between all ALS patients and healthy controls

^b^Fisher’s exact test refers to comparison between all groups (ALS patients with spinal and bulbar onset and healthy controls)

^c^Mann-Whitney-*U*-test refers to comparison between all ALS patients and healthy controls or between ALS patients with spinal and bulbar onset.

^d^Kruskal-Wallis analysis of variances on ranks between healthy controls, ALS patients with bulbar and spinal onset.

^†^Post-hoc comparison for *p*<0.05 using Mann-Whitney-*U*-test: *p*<0.001 for ALS patients with spinal onset compared with healthy controls.

^e^ALSFRS-R, revised ALS Functional Rating Scale (maximum score 48, falling with increasing physical impairment) [9]

^f^ECAS, Edinburgh Cognitive and Behavioural Amyotrophic Lateral Sclerosis Screen (maximum score 136, falling with cognitive decline) [10]

NA, not applicable.

The diagnosis of ALS was made according to the revised El Escorial diagnostic criteria [11] by board-certified neurologists specialized in motor neuron disease. Exclusion criteria were all other neurodegenerative diseases such as Parkinson’s Disease and other Parkinsonian syndromes, any history of psychiatric disorders, abnormal vision, or severe hearing impairment. None of the patients had severe respiratory deficits or was treated with non-invasive ventilation. In addition, none of the ALS patients had any clinically suspected form of dementia and failed to meet criteria for frontotemporal lobar degeneration (FTD) according to recently proposed guidelines [12]. None of the controls had any clinically significant medical condition, psychiatric illness, hearing impairment, or visual acuity abnormalities.

### Cognitive assessment

For cognitive screening, the German version of the Edinburgh Cognitive and Behavioral ALS Screen (ECAS) was used, assessing ALS specific cognitive functions, such as language, verbal fluency and executive functioning, and non-ALS specific functions, such as memory and visuospatial perception. The maximum total score is 136 with decreasing scores indicating lower cognitive performance. The cut-off score for clinically significant cognitive impairment is lower than 92 [10], and 12 patients did not reach this cut-off score (see also Table 1).

### Recording of eye movements

Eye movements were measured using the video-oculography EyeSeeCam® device (EyeSeeTec GmbH, Fürstenfeldbruck, Germany) that records binocular eye positions synchronously with 0.02° spatial resolution at a temporal sampling rate of 220Hz [13]. Eye movements were acquired in our oculomotor laboratory [14–17]. All participants were comfortably seated with their eyes facing a white hemi-cylindrical screen in a softly lit and acoustically shielded environment. To minimize confounding head motion, subjects' heads were stabilized by an adjustable chin rest, such that the vertex was at the center of the hemi-cylindrical screen, resulting in an eyes-to-screen distance of approximately 150 cm (depending on the subjects' head anatomy). The hemi-cylindrical screen had pairs of vertically adjacent red and green light- emitting diodes (*d* = 0.3°) placed equidistantly every 5° up to ±20° in horizontal and up to ±15° in a vertical direction, which served as targets and were invisible when not lit. A mirror galvanometer was mounted above the subject’s head to elicit smooth pursuit eye movements by moving a red laser spot (*d* = 0.15°) across the screen in horizontal (range ±20°) and vertical direction (range ±15°). Loudspeakers were placed adjacent to the screen in 90° to the left and right of center.

*Smooth pursuit eye movements* were tested in horizontal direction by sinusoidal oscillation with *f* = 0.375Hz (12 cycles = 32s) resulting in a peak target velocity of 47.1°/s. Subjects were instructed to track the moving spot as accurate as possible [18].

*Visually guided reactive saccades* were pseudo-randomly elicited in horizontal (32 target steps, i.e., three times of ±5, ±10°, ±15,° ±40° and 4 times of ±20°, targets within range ±20°, 92.8s acquisition time) and in a vertical direction (36 target steps, i.e., 4 times of ±5°, ±10°, ±15°, ±30° and two times ±20°, targets within range ±15°, 93.6s acquisition time) by lighting red light emission diodes, so that each target step proceeded with the previous step. The targets were presented for 2.9s on average (range 2.1–3.5s) in a horizontal and for 2.6s (2.1–3.5s) in a vertical direction. Subjects were asked to re-fixate to the new target as quickly and accurately as possible but to withhold their gaze shift until the next target appeared [14].

*Delayed-saccades* were tested by pseudo-randomly presenting a new red additive target after 1.7s on average (range 1.1–2.3s) at 5, 10, 20, and 40° horizontal positions (either 8 trials to the left and right), until an acoustic ‘go’ cue was given. A cue was pseudo-randomly presented acoustically after the new additive target onset. Subjects were asked to withhold their reaction to the new additive target until the cue was sounded. Each target step proceeded with the previous step [19].

*Anti-saccades* were tested by pseudo-randomly presenting green targets (either 8 trials to the left and right) at ±5, ±10 and ±15, and ±20° eccentric horizontal positions after 2.6s on average (range 2.1–3.0s). A training session of five runs was administered prior to the anti-saccade condition. Participants were requested to instantly initiate a gaze shift towards the mirror (opposite) position of the new target [19].

*Rapid alternating voluntary gaze shifts* were evoked in horizontal and vertical directions by requesting subjects to saccade for 30s as rapidly as possible back and forth between two steady green targets arranged symmetrically about the primary direction with 20° horizontal or vertical angular separation [15].

### Analysis of eye movements

An interactive MATLAB® (The Mathworks Inc., Natick, MA, USA)-based in-house software package *OculoMotor Analysis* [14,15,18–20] was used for analysis of eye movement recordings. Preprocessing included noise reduction by low pass filtering (*f*<30Hz), cross-talk suppression, calibration, and deletion of artefacts (blinks, corrupted signals). The calibration procedures require the subject to track the target which was given for horizontal and vertical ‘slow’ sinusoidal single-spot target oscillation (mirror galvanometer) to map the non-calibrated orthogonalized ‘raw’ data from the EyeSeeCam® device with respect to the ‘true’ orthogonalized eye position (horizontal range ±20°; vertical range ±15°, *f* = 0.125 Hz). Neither the patient group nor the control group exhibited systematic differences between the right and the left eye. Therefore, the binocular recording was merged into a cyclopean signal averaging the monocular recordings [19]. All records were visually inspected for quality assurance. From the eye movement paradigms examined as described above the following parameters were extracted:

- *Smooth pursuit eye movement* yielded two measures: 1) the smooth pursuit gain as a function of smooth eye velocity/target velocity, and 2) the phenotype (anticipatory or catch-up saccades) of saccades interrupting smooth pursuit [18,19]. To automatically classify the type of smooth pursuit error, the amplitudes of all saccades were accumulated. Hence, a negative total accumulated saccade amplitude sum corresponds to the predominance of back-corrections and catch-up saccades. In synopsis with a reduced gain as the quantitative marker for evident smooth pursuit pathology, a negative amplitude sum indicates a deficient smooth pursuit component that may be attributed to a disturbed ponto-cerebellary saccade generation network [18]. A positive amplitude sum indicates predominant anticipatory saccades during pursuit that are related to a cortical (frontal) ‘top-down’ pathology of eye movement control.
- *Visually guided reactive saccades* (VGRS) were characterized by the primary saccade gain, peak eye velocity (each for horizontal, up, down), the latency (horizontal, vertical) according to [19,21], and the rate of saccadic intrusions (horizontal). Saccadic intrusions were examined for horizontal reactive saccades and computed as the accumulated amplitude of saccades excluding the primary saccades divided by the considered time interval (i.e., ‘prevalence’ or rate of saccadic intrusions in degrees per second). Saccades <2° amplitude were excluded from the saccadic intrusions rate analysis.
- For *delayed-saccades* and *anti-saccades*, percentage of errors, i.e., saccades before cue and pro-saccades, were obtained as described previously [19].
- *Rapid alternating voluntary gaze shifts* exceeding 10° saccade amplitude were counted for both tasks, i.e. in the horizontal and vertical direction. The number of such shifts was arithmetically averaged for horizontal and vertical directions since the outcomes of both direction were considered to be basically similar [15,18].

### Identification of eye movement features that qualify for a sequential staging approach

To qualify eye movement control as a potential marker for identifying a sequential pattern of oculomotor decline, the oculomotor parameter in question must show a new quality of pathology rather than confirmation of an already existing pathology. According to the eye movement parameters described above, this approach requires the identification of those oculomotor deficits that comprise a characteristic phenotype of function, which can be traced back to a specific brain network ALS-related pathology that underlies these oculomotor deficits. Subsequently, these manifestations of oculomotor decline may be analyzed with respect to their pattern of appearance and in relation to distinct clinical features in the ALS patients tested.

Based on these assumptions, the following parameters qualify for staging: 1) Error rates in the delayed- and antisaccade task; 2) number of rapid alternating voluntary gaze shifts as well as 3) intrusions during fixation, and anticipatory behavior during smooth pursuit eye movement (SPEM). Performance pathology in these parameters may be ascribed to deficits of executive control [15,18]. 4) Reduced peak eye velocity is attributed to a defective triggering in the paramedian pontine reticular formation (PPRF) and rostral interstitial nucleus of the medial longitudinal fascicle (riMLF) [7]. 5) The appearance of catch-up saccades is seen as a specific sign of a disturbance within the ponto-cerebellary network [22].

To classify the pathologic character of these parameters on an individual patient level, we considered values ‘outside’ the 1.5 times interquartile range as abnormal. Based on our experience in different patient populations and a vast database of healthy controls [14,15,18–20], this approach appears to be specific but less sensitive; however, it prevents false positive staging errors.

Given this approach and in view of the pattern of sequential progression of pTDP-43 pathology in ALS [2] we used a staging system consisting of two stages as follows: stage 1 cases revealed only deficits of executive control, and stage 2 cases displayed deficits of brainstem function or within the precerebellary pontine network in addition to deficits of executive control.

### Statistical analysis

The MATLAB® based ‘Statistics Toolbox’ was used for all statistical data analyses. Data on subject characteristics and eye movement parameters were described using the median (and interquartile range). In accordance with previous studies [14,15,23], we used non-parametric interference statistics to compare the eye movement parameters between the cohorts because we cannot assume a normal distribution of the oculomotor parameters. Statistical interference between groups was analysed using Fisher’s exact test for categorical variables or Wilcoxon-Mann-Whitney-*U*-test and Kruskal-Wallis analysis of variances on ranks for continuous variables, respectively. In case of three groups (ALS patients with spinal and bulbar onset, healthy controls), the Kruskal-Wallis analysis of variances on ranks was followed in the event of significance (*p*<0.05) by Wilcoxon-Mann-Whitney-*U*-test. Spearman's rank order correlation coefficient was used to study possible relationships between eye movement parameters and clinical scores for the patient group. The resulting *p*-values were corrected for multiple comparisons (family-wise error correction). All statistical tests were 2-sided with *p*<0.05 indicating statistical significance.

## Results

### Deficits of eye movement control in patients with ALS

Results of eye movement analysis in the sample of all ALS patients (*N* = 68) compared to all controls (*N* = 31) and the comparisons between ALS patients with spinal (*N* = 50) and bulbar onset (*N* = 18), respectively, and all controls are summarized in Table 2. Parameters corresponding to executive eye movement control were severely altered in patients with ALS compared to controls. In the delayed and anti-saccade task, patients presented a high distractibility with pronounced difficulties in suppression of unwanted gaze shifts by frequently moving their eyes towards the target (pro-saccade or gaze shift before ‘cue’) (*p*<0.001). Many of these directional errors were rapidly corrected, indicating that the subjects had no difficulties in understanding the task. The ability to perform self-initiated gaze shifts (reduced number of rapid alternating voluntary gaze shifts) between two fixed targets was considerably deficient in ALS patients (*p* = 0.003). SPEM performance revealed no statistical differences between patients and controls, although at the individual level some patients presented with frequent anticipatory saccades interrupting smooth pursuit and considerable ‘catch-up’ saccades correcting for reduced SPEM gain. Abnormally large and frequent saccadic intrusions were observed during fixation when ALS patients, in comparison to controls, awaited a new target position. Reactive saccades revealed primary saccade gains that were similar to those of controls; however, peak eye velocities, especially in horizontal and upper directions (*p*<0.02), were significantly slowed. Moreover, saccade latencies were significantly prolonged in patients compared with controls (*p*<0.02). Remarkably, ALS subgroup analysis (spinal vs. bulbar onset) revealed no significant differences in the investigated oculomotor parameters with an exception of the upward VGRS gain which indicated a tendency for more hypometric saccades in upper direction in bulbar onset compared to spinal onset (*p* = 0.035).

**Table 2:** Video-oculographic data of the 99 subjects. Data are depicted as median (interquartile range), min-max; post-hoc comparisons (for pair-wise statistical interference between ALS patients spinal onset, ALS patients bulbar onset and controls) reaching statistical significance are indicated as upper scripts ^†^ and ^#^ (see subsequent footnotes for details).

	Healthy controls (*N* = 31)	ALS, All (*N* = 68)	*p*-value ^a^	ALS, spinal onset (*N* = 50)	ALS, bulbar onset (*N* = 18)	ANOVA *p* ^b^
**SPEM gain**, %	**90** (53–95), 22–100	**82** (59–92), 13–104	0.303	**81** (60–92), 13–104	**84** (55–92), 21–102	0.557
**SPEM accumulative saccade sum / °**	**6** (0–142), -3–317	**4** (-6–58), -364–398	0.140	**7** (-6–56), -168–398	**1** (-3–65), -364–321	0.334
**VGRS (horiz.) gain** ^c^, %	**88** (85–91), 77–96	**88** (84–91), 73–96	0.661	**88 (84–91), 73–96**	**86 (82–90), 76–93**	0.398
**VGRS (down) gain** ^c^, %	**93** (89–100), 76–110	**92** (84–97), 61–113	0.119	**92** (84–95), 66–113	**93** (85–97), 61–106	0.267
**VGRS (up) gain** ^c^, %	**78** (75–83), 57–91	**77** (68–83), 46–95	0.255	**79** (71–84), 46–95	**72** (64–78), 53–90^**†#**^	**0.045**
**VGRS (horiz.) latency** ^d^ / ms	**233** (214–252), 199–300	**248** (224–269), 182–343	**0.049**	**251** (227–268), 182–343	**237** (214–273), 193–327	0.054
**VGRS (vert.) latency** ^d^ / ms	**241** (230–270), 214–320	**265** (244–291), 191–400	**0.011**	**268** (245–291), 191–400^**†**^	**252** (241–275), 223–315	**0.022**
**VGRS (horiz.) peak eye velocity** ^**e**^ **/** °/s	**430** (398–453), 309–508	**399** (358–435), 207–534	**0.018**	**399** (359–435), 207–532	**400** (349–432), 224–534	0.059
**VGRS (down) peak eye velocity** ^**e**^ **/** °/s	**384** (342–450), 281–511	**368** (334–411), 171–541	0.121	**367** (330–396), 171–541	**389** (348–419), 260–514	0.199
**VGRS (up) peak eye velocity** ^**e**^ **/** °/s	**429** (391–494), 289–573	**382** (331–443), 99–601	**0.006**	**374** (328–443), 237–591^**†**^	**391** (341–444), 99–601	**0.021**
**VGRS, intrusion rate** ^**f**^ **/** °/s	**1.2** (1.0–1.7), 0.5–2.7	**1.7** (1.1–2.3), 0.5–3.5	**0.032**	**1.7** (1.0–2.4), 0.5–3.5	**1.7** (1.4–2.1), 0.9–3.2	0.099
**Delayed-saccades error rate** ^g^, **%**	**6** (6–12), 0–25	**19** (13–44), 0–100	**<0.0001**	**19** (12–38), 0–94^**†**^	**19** (12–56), 0–100^**†**^	**<0.001**
**Anti-saccades error rate** ^**h**^, **%**	**19** (6–25), 0–75	**41** (19–67), 0–100	**<0.0001**	**44** (25–67), 0–100^**†**^	**38** (6–75), 0–100^**†**^	**<0.0001**
**Number of voluntary gaze shifts** ^i^	**58** (48–72), 33–98	**50** (37–59), 15–82	**0.003**	**50** (42–59), 20–78^**†**^	**44** (26–60), 12–82^**†**^	**0.011**

^a^Mann-Whitney-*U*-test between healthy controls and all ALS patients

^b^Kruskal-Wallis analysis of variances on ranks (ANOVA) between healthy controls, ALS patients with bulbar and spinal onset. Post-hoc comparison in the event of ANOVA *p*<0.05 using Mann-Whitney-*U*-test: *p*<0.05 for ALS patients with spinal onset compared with healthy controls^†^ and *p*<0.05 for ALS patients with bulbar onset compared with controls^†^; *p* = 0.035 for ALS patients with bulbar onset compared with ALS patients with spinal onset^#^

^c^Gain of VGRS aimed at targets of 20° eccentricity obtained by linear fitting saccade amplitudes as a function of target steps

^d^Latencies of VGRS with respect to primary saccade onset

^e^Peak eye velocity of VGRS aimed at targets of 20° eccentricity obtained by non-linear interpolation along the main sequence

^f^Saccadic intrusions (>2°) excluding the primary saccade, computed as the sum of saccades within VGRS acquisition time

^g^Erroneous responses (saccades before cue)

^h^Erroneous responses (pro-saccades)

^i^Saccades (>10°) counted within 30s

### Eye movement characterization on single patient level

The subsequent results describe patients’ oculomotor performance on single patient level. Values ‘outside’ the median±1.5 times interquartile range of normal controls’ performance were regarded as pathologic.

### No deficits in eye movement control

The eye movement performance in 30 ALS patients was similar to that in all controls. None of the investigated eye movement parameters were outside the 1.5 times interquartile range of the control group. Comparison between this group of patients and controls showed no statistically significant effect in any oculomotor parameter (Table 2).

### Impaired executive oculomotor control (classified as stage 1)

**Fig 1:**
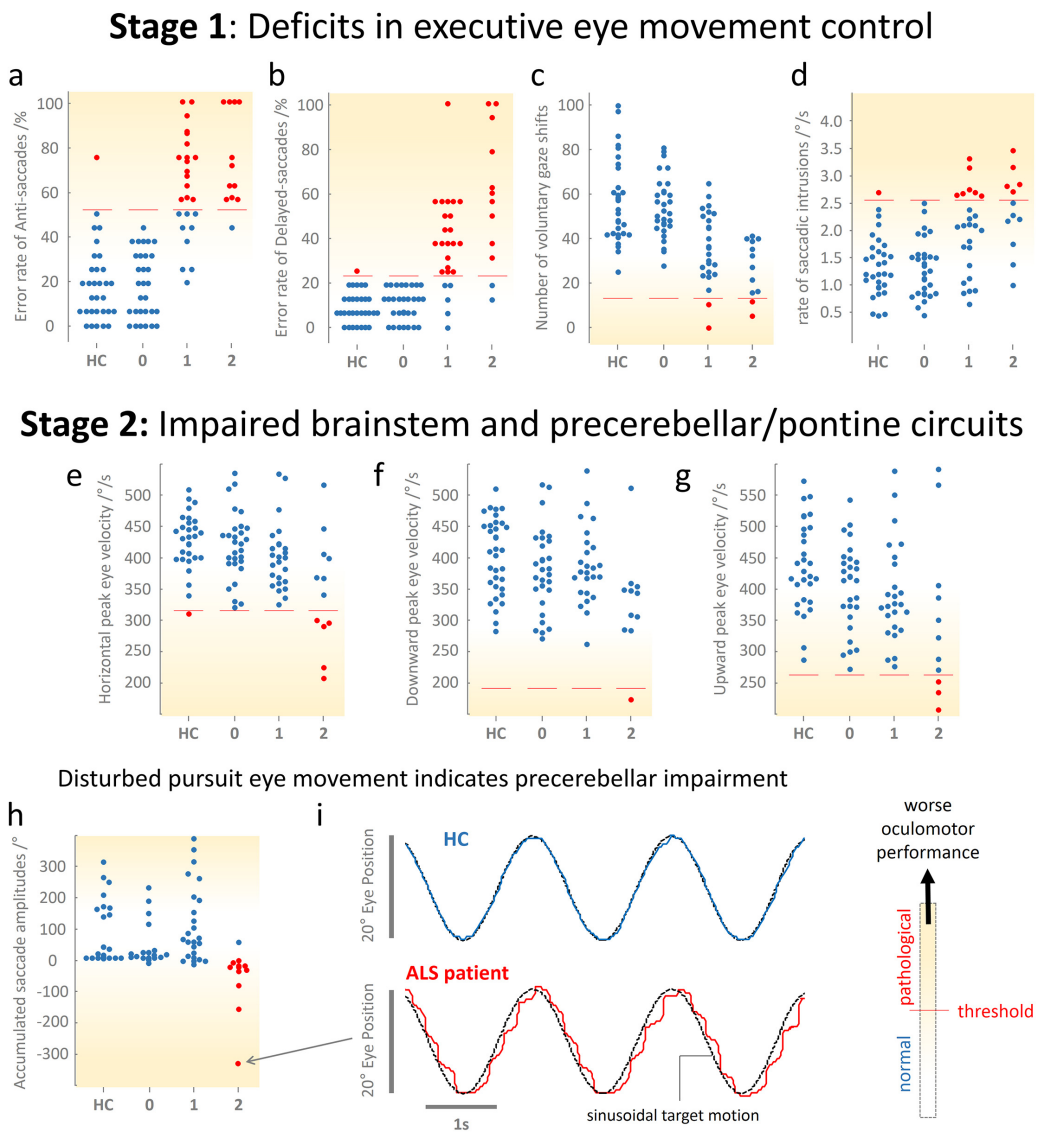
Categories of oculomotor deficits used for the proposed staging scheme. Results of eye movement measures used to characterize the sequential progression of oculomotor impairment in ALS patients. Scatter plots (**a**—**h**) showing the distribution of the respective eye movement parameter in healthy controls (**HC**) and ALS patients falling into stage **0**, **1**, and **2**. The **shading intensity** in each plot indicates the increasing deficit in eye movement control raging from normal (**white**) to severely impaired performance (**yellow**). Normal outcomes are shown as **blue dots**, values considered as pathological are depicted as **red dots**. The threshold (**red line**) was computed from the control group and is defined as upper quartile plus 1.5 times the interquartile range (IQR) or lower quartile minus 1.5 times IQR, respectively. (**a**—**d**) Eye movement parameters reflecting executive control. (**e**—**f**) Peak eye velocities obtained from visually guided reactive saccades indicating malfunctions of the brainstem oculomotor nuclei and the underlying network. (**h**) Accumulated sum of saccades interrupting smooth pursuit. Negative values together with a reduced pursuit gain (not shown, for details see text) indicate the prevalence of catch-up saccades. With increasing positive values, smooth pursuit becomes disrupted due to saccadic intrusions but the ability to perform perfect pursuit remains preserved. (**i**) Sample recordings of smooth pursuit (**blue traces**) in one representative healthy control subject (HC, **upper row**) and one patients with ALS (**lower row**) during tracking of a horizontal sinusoidal (*f* = 0.375Hz, amplitude ±20°) target motion (**black dashed traces**). Whereas the control subjects performed perfectly, the patient presented a substantial gain lag that is compensated by almost periodical catch-up saccades in order to bring the eye back onto the target, considerably indicating an impaired precerebellar pontine circuit.

Each of the 25 patients with ALS classified at stage 1 displayed impairments (outside the 1.5 times interquartile rage) in at least one executive oculomotor domain compared with controls. For the group comparison we found increased error rates in delayed- (*p*<0.001) and anti-saccades (*p*<0.001), reduced number of rapid voluntary gaze shifts (*p*<0.001), or increased incidence of saccadic intrusions (*p* = 0.004), respectively. Fig 1 (upper row) shows the distribution of eye movement measures associated with executive control. Saccades that interrupted episodes of perfect smooth pursuit predominantly *anticipate* target motion; no predominant pattern of catch-up saccades was observed, thereby indicating no considerable damage to brainstem and pontocerebellary network related oculomotor functions. Nevertheless, in all patients assigned to stage 1, peak eye velocities (>238°/s at 20° saccade amplitude) and primary saccade gains were similar to those of controls, although a tendency towards prolonged latencies was detectable.

### Involvement of brainstem and pontocerebellary network related oculomotor functions (classified as stage 2)

**Fig 2:**
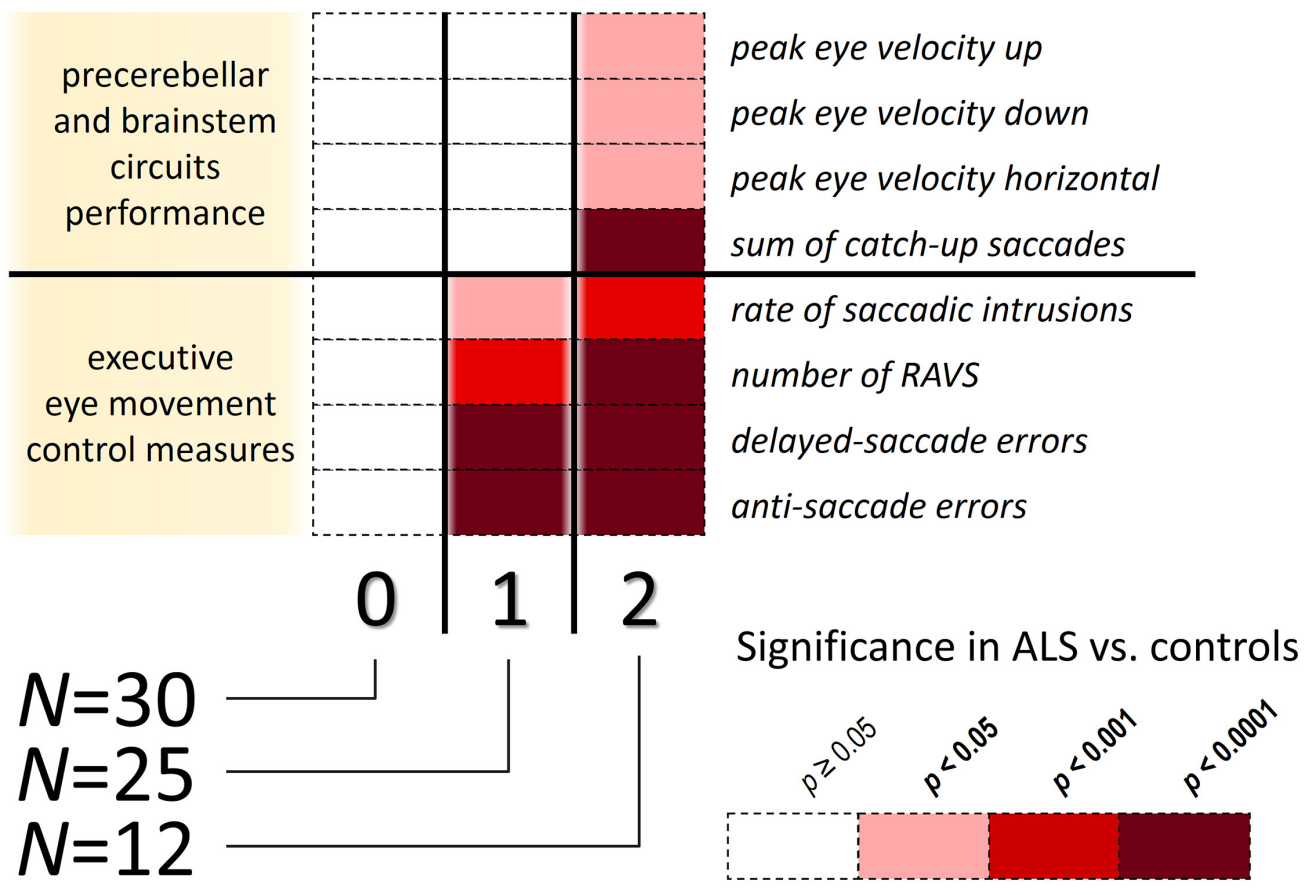
Sequential progression of impaired eye movement control in ALS. About the half of the investigated patients with ALS were (**0**) without any susceptibilities in eye movement control (Stage ‘0’), the others with an exception of one patient fall into two subgroups. Given the consistency of this findings, the two subgroups can be arranged to show disease progression based on (**1**) deficits in executive eye movement control (Stage 1) and (**2**) brainstem and pontocerebellary network related oculomotor deficits comprising impaired brainstem function or disturbances in the precerebellar pontine network that paralleled gradually worsening executive oculomotor performance (Stage 2). The level of statistical significance is color coded and indicates comparison between the staged ALS patients and healthy controls. Note, that one out of 68 subject did not follow the proposed staging scheme. RAVS, rapid alternating voluntary gaze shifts.

A total of 12 ALS patients were assigned to stage 2 because they showed both executive dysfunctions similar to stage 1 individuals and exhibited in addition frequent catch-up saccades and/or deficient maximum peak velocity (Fig 1, center and lower row). In one case, saccadic intrusions temporarily co-occurred during pursuit, but the pattern indicated the presence of periodical catch-up saccades that typically *correct* the defective smooth pursuit eye movements (Fig 1, lower panel). Five of the ALS patients also revealed ‘slowed’ saccades, i.e., their peak eye velocity was significantly reduced, note that 3 of them showed spinal and 2 bulbar onset. Moreover, executive control pathology gradually worsened compared to those patients in stage 1. Further on, the gains of primary saccades were similar to that in controls and in patients classified at stage 1, whereas the latencies were significantly prolonged (*p*<0.006). The staging system proposed here on the basis of eye movement recordings is illustrated in Fig 2. Only one out of 68 patients with ALS presented a feature typical of stage 2 in the absence of stage 1 pathology; this was the only patient that did not fit into the proposed staging scheme.

### Correlations of eye movement parameters with clinical scores and neuropsychological assessment

Significant correlations between the ALSFRS-R clinical score and all of the executive eye movement control parameters, i.e., delayed- (*r* = 0.75, *p*<0.001) and anti-saccades errors (*r* = 0.77, *p*<0.001), the number of voluntary gaze shifts (*r* = -0.62, *p*<0.001), and saccadic intrusion rate (*r* = 0.54, *p*<0.001) were found. The ECAS as a sensitive test for frontal function in ALS revealed tight correlations with delayed- (*r* = -0.51, *p*<0.001) and anti-saccades’ errors (*r* = -0.41, *p*<0.001) and with the number of voluntary gaze shifts (*r* = 0.47, *p*<0.001), but not with the parameters that code for brainstem and pontocerebellary network pathology. There was no significant correlation in ALS patients between any of the oculomotor parameters and both disease duration as well as the initial symptom onset (bulbar or spinal onset). We did not find any clues for a reduced peak eye velocity linked to the initial symptom onset, such as bulbar or spinal onset. Finally, patients’ assignment to the staging including no oculomotor deficits and stages 1–2 significantly correlated with clinical ALSFRS-R score (*r* = -0.39, *p*<0.001).

## Discussion

The present study on eye movement control in a large cohort of ALS patients shows that oculomotor deterioration in motor neuron disease parallels the neuropathological staging of the disease [2]. For this purpose, we 1) systematically characterized eye movement deficits in ALS, as examined by video-oculography, 2) assigned these deficits to the network pathology in ALS based on current knowledge about the neuropathological pattern of disease progression and the biology of eye movements, and 3) looked to see if pathology in oculomotor networks itself follows a sequential pattern, thereby making possible an oculomotor staging protocol that not only reflects the neuropathological staging of the disease but also may be able to serve as a marker of disease progression.

In accordance with existing literature [3–5], we found a broad spectrum of oculomotor deficits in the 68 ALS patients in comparison to 31 matched controls. The characteristics of oculomotor dysfunction on single patient level and its sequential appearance enabled us to classify eye movement pathology into two stages. In stage 1, only deficits in executive control of eye movements are involved that are ascribed to frontal- and prefrontal-brain pathology and to its descending cortico-striatal pathways. Stage 2 indicates the transition from neuropathological stage 1 to stage 2, as the pulse-generating part of the brainstem circuitry for saccade generation becomes impaired. With respect to spinal and bulbar onset ALS, there was no significant difference in oculomotor performance at group level apart from upward VGRS gain which is not part of the staging algorithm, hence the sub-groups were merged for the oculomotor staging.

### Oculomotor deficits in ALS

The most prominent oculomotor alterations were observed in those oculomotor parameters that are assigned to frontal lobe impairment (37 of 68 patients). A similar pattern with joint occurrence of elevated error rates of antisaccades and delayed saccades has been described in patients with FTD [24,25], progressive supranuclear palsy (PSP) [7,18], and Alzheimer’s disease [25,26] but also in ALS patients [27,28]. These deficits have been attributed to dysfunction of the dorsolateral prefrontal cortex (DLPFC), but also the frontal eye fields (FEF) and the supplementary eye field (SEF) can exert contextual control over saccade generation [29–31].

Rapid alternating voluntary gaze shifts are a relatively new and, up to now, not routinely used parameter that also codes for a deficit in saccades initiation and may also be attributed to fronto-striatal pathway dysfunction inasmuch as it has proved to show a high correlation to cognitive decline and especially frontal lobe dysfunction [19].

For the majority of ALS patients examined, the pulse-generating part of the brainstem circuitry for saccade generation [6] was unimpaired. Nevertheless, 12 ALS patients showed a particularly high degree of impairment with interrupted smooth pursuit of the cerebellar type, and five additional patients displayed severely reduced maximal saccades velocity (i.e., gaze palsy). Notably–apart from one patient–these deficits only occurred in addition to the executive oculomotor impairment described here above and *not* as an exclusive oculomotor feature in these patients.

Gaze palsy is the eponymous finding in PSP [32] and is seen as a specific phenomenon associated with pathology in the raphe interpositus nucleus [7]. It has also been described previously in ALS. Donaghy et al. [28] even suggested that there might be a “PSP variant” of ALS, because until recently affection of the raphe interpositus nucleus was not considered to be a frequent feature of ALS. Furthermore reduced peak eye velocity was equally-distributed over spinal and bulbar onset ALS patients, of those 5 patients with significantly reduced peak eye velocity 2 showed a bulbar and 3 showed a spinal onset. Thus we cannot confirm that slow saccades are an eponymous feature of bulbar onset MND as suggested by Donaghy et al. [28].

Reduced SPEM gain either results from an involvement of the ponto-cerebellar network controlling the pursuit motor signal or from fronto-striatal dysfunction leading to attentional deficits and interruptions of SPEM by the release of inappropriate saccades (intrusions) [18]. Although reduced SPEM gain in ALS has been described before, the data as well as the discussion about its underlying pathology are heterogeneous [28,33,34]. By inventing an algorithm that is capable of fractionating SPEM pathology into two predominance patterns (catch-up saccades as a sign of ponto-cerebellary impairment, on the one hand, and anticipatory saccades as well as intrusions, on the other), we managed to isolate a group of 12 ALS patients with a predominantly ponto-cerebellar type of SPEM pathology. We thus conclude that both types of SPEM pathology can occur in ALS. Although the pattern of anticipatory saccades and intrusions into SPEM is the more frequent finding, appearance of cerebellary SPEM pathology in ALS is of utmost interest inasmuch as it serves as a ‘red flag’ for deficits within ponto-cerebellary pathways. As addressed by Moss et al [35] oculomotor abnormalities are more frequent in patients with ALS than in similar-aged controls. Yet, it has to be taken into account that there is a considerable overlap of oculomotor abnormalities in ALS with those described in other neurodegenerative diseases such as Parkinsonian syndromes [32]. Hence, only in conjunction with the clinical hallmarks of an upper and lower motor neuron disease, accurate examination of oculomotion may contribute to the clinical diagnosis of ALS.

### Oculomotor staging

With respect to our results that basically confirm recent literature regarding abnormal eye movements in ALS, oculomotor decline can be categorized into two stages that are aligned to a stage-related neuropathology in ALS. The here proposed oculomotor staging model in ALS consists of two stages, stage 1 (confined to abnormal executive control of oculomotion) and stage 2 (additional occurrence of either catch-up saccades and/or gaze palsy). It is worth emphasizing that we made it mandatory that all patients who qualified for stage 2 also had deficits in at least one stage 1-related oculomotor parameter. If they failed to do so they were classified as not fitting the oculomotor staging model. All but one of the 68 ALS patients tested could be categorized within our staging model of oculomotion, and this, in turn, emphasizes its credibility as operationalized.

#### Oculomotor stage 1

Executive control of oculomotion is regarded as a function of the frontal and prefrontal cortex and its descending pathways. The supplementary eye field (SEF, Broadman area 6) is part of the agranular frontal neocortex (Broadman areas 4 and 6) where the earliest detectable lesions in ALS develop [2,8]. [31] have shown that the SEF can exert contextual executive control over saccade generation. Sharika et al. [36] recently reported that the SEF is sensitive to response conflict and errors and that it implements context-based behavioral adjustments. Thakkar et al. [37] concluded that the SEF and pre-SEF are part of a network that is involved in rapidly inhibiting and changing an eye movement. Hence, even early in the course of ALS (neuropathological stage 1), patients may show impaired executive control over saccades. With further disease progression to prefrontal areas as well as to parieto-occipital cortical areas, more regions regarded as crucial for executive oculomotor control, e.g., the DLPFC, inferior parietal lobe (= LIP) and the parietal eye field [29] become involved and may contribute to the oculomotor deficit seen here. But even ALS stage 3 involvement of the striatum may contribute to this type of oculomotor pathology [16]. We also have to acknowledge that there is no appropriate oculomotor parameter that can distinguish between these multiple regional influences–at least not in a cross-sectional approach. Nonetheless, we think that the quantitative decline of executive oculomotor parameters from our proposed oculomotor stage 1 to stage 2 can be regarded as an indicator for the progression of the pathology.

#### Oculomotor stage 2

In ALS neuropathologic stage 2, the reticular formation and pontine precerebellar nuclei of the lower brainstem become involved [2]. Disturbance of excitatory burst neurons in the PPRF and in the riMLF are the main mechanisms for slowing of saccadic maximum velocity, a feature that is clinically defined as gaze palsy [7]. Both clinical experience and animal experiments [22,38] indicate that the cerebellar vermis, the vestibulo-cerebellum, and the corresponding pontine nuclei are vital elements of the SPEM pathway [22]. For this reason, the occurrence of reduced saccadic velocity and/or the appearance of catch-up saccades in a subgroup of our ALS patients point to the affection of the reticular formation and/or the pontine precerebellar nuclei and, hence, may serve as a marker for the transition from neuropathologic ALS stage 1 to stage 2.

### Correlation of oculomotor staging and the clinical phenotype

The highly significant correlation of the neuropsychological scores with the executive oculomotor parameters adds to the idea that these qualities of oculomotor function are closely related to a predominantly prefrontal and frontal cortical deficit as well as its descending pathways. Hence, our data confirm the repeated notion of a joint occurrence of oculomotor decline and cognitive impairment [28,39]. As ALS and FTD are clinical manifestations within a pathological spectrum [8], it is important to emphasize that none of the patients fulfilled the diagnostic criteria for FTD. If a possible presence of a subclinical behavioral variant FTD may contribute to the oculomotor performance in ALS will have to be further investigated [40].

Moreover, the ALSFRS-R score correlated significantly with both executive parameters of eye movement control and the oculomotor staging, thus supporting the usefulness of oculomotor measurement and oculomotor staging in particular as a marker of disease progression in ALS. Hence oculomotor investigation may add to the in vivo monitoring of the disease stages in ALS in a multimodal setting in combination with diffusion tensor imaging analyses [41]. Burrell and co-workers [5] found that early saccades during visually guided reactive saccades, i.e. saccades with abnormally short latencies, were more present in patients with ALS regardless of the clinical phenotype; however, the rate of early saccades did not significantly correlate with motor or functional impairment. The discrepancy between our results and the study of Burrell and co-workers [5] may be explained by methodological differences, as Burrell et al. investigated the distribution of exclusively early saccades as a measure for executive oculomotor function rather than an accumulated sum of saccadic intrusions.

The list of vulnerable nerve cells that develop ALS-associated pathology includes almost all cell types (e.g. cholinergic, glutamatergic, GABA-ergic) including motor- and non-motor neurons [8]. Imaging studies using positron emission and magnetic resonance tomography indicated a predisposition of inhibitory inter-neuronal cell populations including cholinergic interneurons [42,43]. Nevertheless, only a combined examination of oculomotor control and functional imaging will be able to provide information to what extent specific circuits may contribute to the oculomotor phenotype in ALS.

### Limitations of the study

The study has several limitations. First, a large proportion of patients (*N* = 30) failed to show any oculomotor deficit that could pass for definite pathology on an individual level. In this context, it is imperative to realize that oculomotor investigation is capable of eliciting correlates of disturbed brain networks; however, it is not yet possible to attribute these correlates to a specific disease entity because a multitude of neurodegenerative as well as non-degenerative diseases, and even normal aging, can exhibit a similar oculomotor phenotype [15,19,44,45]. Thus, to avoid false positive categorization, it was absolutely essential to opt for rigorous thresholds to the disadvantage of sensitivity (Fig 1).

Second, executive oculomotor control with an unchanged oculomotor phenotype may be disturbed by pathology in brain regions that become sequentially involved from neuropathologic stage 1 to 3. If quantitative changes of executive deterioration are to serve as a transition marker between these stages, such changes will have to be demonstrable and proved in a longitudinal setting. Although oculomotor decline possibly mirrors the neuropathological progression, the time lag between neuropathological involvement of a distinct brain region and a potential loss of function within this region is not known, especially because the conceptional design of the oculomotor staging was not autopsy-confirmed.

Third, due to the lack of longitudinal data, our proposed staging scheme might be confounded by different oculomotor phenotypes that are an expression of differences in ALS phenotypes. However, the significant correlations of oculomotor parameters with the cognitive measures (i.e. ECAS score), clinical progression (i.e. ALSFRS-R score), and the fact that stage 2 patients also showed a more severe pathology in stage 1 characteristics support our concept of a potential ‘technical’ marker.

## Conclusion

We could show that oculomotor decline in ALS follows a sequential pattern, initially with disruption of executive eye movement control, followed by affection of the pulse-generating part of the brainstem circuitry for saccade generation. This pattern parallels the recently proposed neuropathological staging scheme for ALS and may serve as a technical marker of neuropathological progression in correlation to the clinical phenotype. In particular, oculomotor examination can indicate the involvement of extra-pyramidal motor frontal and prefrontal cortical areas in oculomotor stage 1 and during the transition from oculomotor stages 1 to 2, when the RF and precerebellary nuclei become involved in the disease process. Further studies on oculomotor performance should include longitudinal data and a subgroup analysis of genetic forms of ALS as well as investigations in (possibly) pre-symptomatic gene carriers. In addition, future investigations in ALS patients should also incorporate correlational analyses with MRI data such as volumetric measures [46] and diffusion tensor imaging [41]. The relation of computer-based MRI measures with deficits of eye movement control in ALS patients is of special interest to gain deeper insights into the pathophysiology of eye movement control in ALS. Longitudinal data might make possible a sub-classification of the cortico-striatal network pathology provided intra-individual shifts to higher stages correlate with a quantitative decline of the corresponding oculomotor deterioration on an individual patient level.
